# Energy-Efficient Gold Flotation via Coarse Particle Generation Using VSI and HPGR Comminution

**DOI:** 10.3390/ma18153553

**Published:** 2025-07-29

**Authors:** Sindhura Thatipamula, Sheila Devasahayam

**Affiliations:** WA School of Mines: Minerals, Energy and Chemical Engineering, Curtin University, Kalgoorlie, WA 6430, Australia; sindhura.t08@gmail.com

**Keywords:** gold flotation, HPGR, VSI, specific energy, energy efficiency, particle size, liberation, collectors

## Abstract

This study investigates the impact of two comminution technologies—Vertical Shaft Impactors (VSI) and High-Pressure Grinding Rolls (HPGR)—on gold flotation performance, using ore samples from the Ballarat Gold Mine, Australia. The motivation stems from the growing need to improve energy efficiency and flotation recovery in mineral processing, particularly under increasing economic and environmental constraints. Despite the widespread use of HPGR and VSI in the industry, limited comparative studies have explored their effects on downstream flotation behavior. Laboratory-scale experiments were conducted across particle size fractions (300–600 µm) using two collector types—Potassium Amyl Xanthate (PAX) and DSP002 (a proprietary dithiophosphate collector) to assess differences in flotation recovery, concentrate grade, and specific energy consumption. The results reveal that HPGR produces more fines and micro-cracks, enhancing liberation but also increasing gangue entrainment and energy demand. Conversely, VSI produces coarser, cubical particles with fewer slimes, achieving higher flotation grades and recoveries at lower energy input. VSI at 600 µm demonstrated the highest flotation efficiency (4241) with only 9.79 kWh/t energy input. These findings support the development of hybrid or tailored comminution strategies for improved flotation selectivity and sustainable processing.

## 1. Introduction

The flotation performance of gold ores is strongly influenced by the characteristics of the feed material generated during the comminution stage. As the mining industry seeks to reduce energy consumption and improve metallurgical efficiency, optimizing the grinding–flotation interface has become a critical focus. In terms of flotation efficiency, the primary method for concentrating gold-bearing minerals depends significantly on the size and surface characteristics of the particles being processed [1]. The comminution process plays a pivotal role in determining these characteristics, influencing downstream flotation performance. Fine grinding is energy-intensive and associated with high operating costs (OPEX), with crushing and grinding consuming about 50% of the total energy in mineral processing operations [2,3].

Among emerging alternatives to conventional crushing and ball milling, High-Pressure Grinding Rolls (HPGR) and Vertical Shaft Impactors (VSI) are increasingly adopted for their energy-efficient profiles and improved liberation characteristics. Recent innovations in energy-efficient comminution devices, such as Vertical Shaft Impactors (VSI) and High-Pressure Grinding Rolls (HPGR), offer potential alternatives to conventional crushing and grinding methods [2]. HPGR operates based on interparticle compression, using counter-rotating rolls to exert high pressures (typically 45–75 bar) on ore particles. This mechanism promotes micro-crack formation, enhancing mineral liberation and reducing overgrinding compared to traditional mills. However, the tendency of HPGR to generate excessive fines can lead to problems such as gangue entrainment and reduced flotation selectivity, particularly when not properly managed. In contrast, VSI crushers use high-speed rotor-driven impacts to fracture particles along natural planes of weakness, resulting in coarser, more angular, and cubical products with fewer ultra-fines. The rock-on-rock or rock-on-anvil impact mechanism in VSI limits excessive fines generation, potentially preserving flotation selectivity while reducing energy requirements. Both VSI and HPGR are integrated into modern gold processing circuits due to their high throughput and lower specific energy consumption, contributing to 10–20% reductions in total energy use [3,4,5]. While both technologies are deployed in various mineral processing circuits, few studies have directly compared their influence on downstream flotation outcomes, particularly in gold processing. The present study addresses this gap by systematically comparing flotation recovery, concentrate grade, flotation efficiency, and energy consumption for HPGR and VSI-processed gold ore at different particle size fractions. Two collectors, PAX and DSP002, were used to investigate the role of reagent chemistry on performance across comminution modes.

This work is motivated by the need to balance liberation and selectivity under rising energy costs and sustainability pressures. By evaluating flotation response as a function of grinding method, particle size, and collector type, the study provides insights that can guide the design of integrated comminution–flotation circuits for gold recovery optimization. Machine learning methods and artificial intelligence techniques have the potential to monitor and optimize flotation processes [6].

### 1.1. HPGR vs. VSI: Key Performance Differences

VSI technology produces consistent and cubical-shaped coarse particles, which may reduce the production of fines and potentially enhance flotation selectivity [7,8,9,10], while HPGR introduces inter-particle breakage and micro-cracks, enhancing mineral liberation and flotation efficiency [11,12,13,14]. Coarse flotation (>150 µm), supported by VSI products, reduces energy intensity, carbon emissions, and water usage, addressing key sustainability challenges [15,16].

This study aims to differentiate itself by providing a comprehensive comparison of these technologies, particularly focusing on their comparative advantages in gold flotation recovery. There have been a few reports on the comparison of VSI and HPGR performance. According to Klingmann [17], HPGR-crushed products yield better leach rates. HPGR tends to produce more micro-cracks and alter surface properties, enhancing the flotation response of certain minerals [18]. Conversely, VSI technology, when applied to coarser fractions, may reduce the production of fines, potentially enhance flotation selectivity and improving concentrate quality [7,8].

### 1.2. Overview of Flotation Efficiency

HPGR-crushed material generally shows higher flotation recovery rates due to finer particle size and increased liberation, especially for complex or finely intergrown ores. However, the presence of fines can lead to lower selectivity if not properly managed. The flotation benefits of HPGR are optimized by adjusting the pressure between the rolls for better liberation, with the optimum HPGR roll pressure for the ores tested being in the range of 45–75 bar [19]. The applied roll pressure (45–75 bar) in HPGR increases micro-cracking and fines generation, which improves liberation but also leads to increased gangue entrainment. This overgrinding reduces selectivity and, in some cases, depresses overall flotation recovery despite improved liberation. This trade-off is more apparent in the finer size fractions.

In contrast, the use of VSI for only the coarser fraction may result in lower overall flotation recovery compared to HPGR, as the coarser particles are less liberated. However, the flotation process may achieve higher selectivity and produce a cleaner concentrate with fewer entrained impurities due to the more uniform particle size and shape produced by the VSI. Gold flotation efficiency depends on its geological origin, composition, and environment. Studies have shown that fine size fractions correlate with increased sulfur recovery, indicating that most gold particles are associated with sulfide minerals [20]. However, the gold grades in those fractions were relatively low compared to those of coarse size fractions (Table 1). Flotation of sulfide minerals depends on the degree of mineral liberation, affecting the flotation recovery and the concentrate grade. The more friable “over-ground” sulfides turn into “slimes”, reducing the flotation recovery. Coarse gold particles have a lower probability of reporting to the concentrate compared to finer gold particles [21,22]. Dilute pulps favor coarse gold flotation with high recoveries realized for sizes between 0.16 mm and 0.71 mm [23].

Although HPGR and VSI have been independently studied in comminution circuits, there is limited research comparing their impact on gold flotation recovery [24,25,26]. The mechanistic understanding of how HPGR-induced micro-cracks influence flotation kinetics remains incomplete, while the potential of VSI-generated coarser particles to improve flotation selectivity has been largely unexplored. This study aims to systematically compare the flotation performance of VSI- and HPGR-derived products, focusing on their influence on particle size distribution, surface properties, and gold recovery efficiency. By bridging this knowledge gap, the findings will contribute to the optimization of comminution circuits for improved gold recovery, providing insights into the trade-offs between energy efficiency, mineral liberation, and flotation performance.

### 1.3. Objectives

The present study aims to study the effects of VSI and HPGR products in terms of liberation, on the coarse flotation recovery and grade of gold ores. Froth flotation on different particle sizes (+300 µm to −600 µm) will be conducted. The influence of collectors on the recovery of gold will also be studied.

## 2. Experimental Section

### 2.1. Sample Preparation

**Figure 1:**
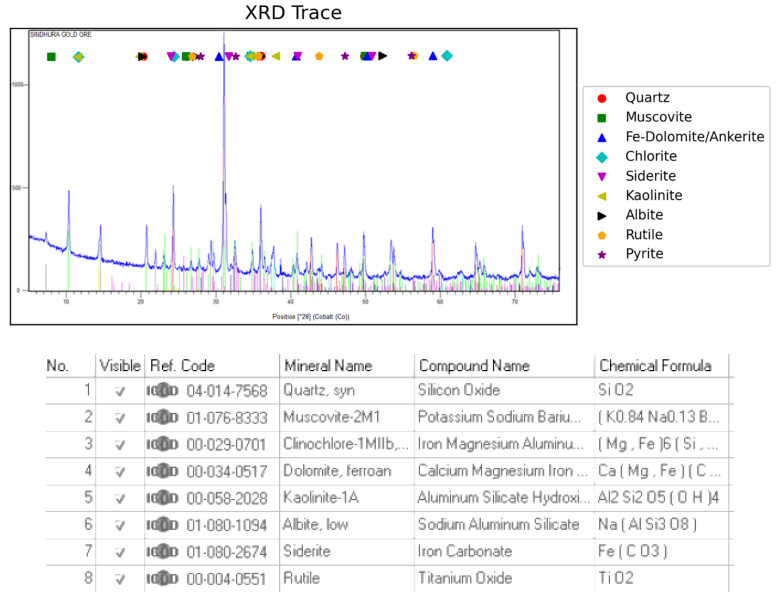
XRD pattern of the raw ore.

About 500 kg of gold ore received from the Ballarat gold mine located in Ballarat, Victoria, Australia, was used in the present study [27]. The total sample used for the study was 60 kg. A total of 5 kg of sample was used for each study. The sample was dried in the oven to remove the moisture. To reduce sample variability, the 500 kg bulk sample from Ballarat was thoroughly homogenized using cone-and-quartering. For each experiment, 5 kg was subsampled under the same protocol. While this process was designed to ensure representative splits, the natural heterogeneity of gold-bearing ores may still influence head grade and mineral distribution across tests. As the study focused on broad comparative analysis without replicates, reproducibility and intra-sample variability were not assessed statistically. This limitation is acknowledged in interpreting the flotation outcomes. The ore characteristics are presented in Table 2. Figure 1 shows the XRD of the raw ore. XRD analysis was performed using a PANalytical X’Pert PRO diffractometer (Malvern Panalytical, Almelo, The Netherlands) with Cu Kα radiation (λ = 1.5406 Å), scanned from 5° to 80° 2θ at a step size of 0.02° and a scan speed of 0.5°/min. XRD is not sensitive to trace levels of gold, especially when gold is present in very fine or disseminated form. The typical detection limit of XRD is insufficient to detect gold in ores where Au is present in ppm levels. This is a common limitation in gold mineralogy studies [28].

### 2.2. Expected Differential Breakage

The breakage behavior of minerals within an ore is influenced by their hardness, brittleness, cleavage, and grain structure [5,29]. Understanding these properties is critical for optimizing comminution efficiency, minimizing overgrinding, and enhancing liberation for improved downstream processing. The expected breakage patterns of the ore constituents (Table 2) are assessed based on their mechanical properties and comminution response [30].

#### 2.2.1. Mineral-Specific Breakage Predictions

Quartz, which accounts for 50% of the sample, has a Mohs hardness of 7 and is highly brittle with conchoidal fracture. It tends to fracture along sharp angular planes, producing fine particles under compressive stress. This enhances liberation but increases the risk of overgrinding [11,31]. Muscovite, present at 18%, has a Mohs hardness of 2.5–3 and is flexible in thin sheets but brittle in bulk. It cleaves along basal planes, yielding flaky particles that resist further size reduction. These platy particles can increase surface area and potentially enhance collector adsorption during flotation [5]. Fe-Dolomite/Ankerite, comprising 11.5% of the sample, has a hardness of 3.5–4 and shows moderate brittleness with rhombohedral cleavage. It is expected to break into medium-sized fragments due to its cleavage planes, reducing the risk of overgrinding [13]. Chlorite (7.9%) is soft (Mohs 2–2.5) and brittle, with a layered silicate structure. It tends to fragment along its sheets, producing fine platelets that may aid flotation but could also contribute to slime generation [32]. Siderite (4.2%) has a Mohs hardness of 3.5–4.5 and is brittle, fracturing along rhombohedral planes. It typically yields coarser fragments, which may reduce the risk of excessive fines but limit liberation efficiency [29]. Kaolinite (3.4%) is very soft (Mohs 2–2.5) and fine-grained. It readily disintegrates into ultrafine particles under stress, contributing to slimes that interfere with flotation selectivity [33]. Albite (3.3%) is moderately hard (Mohs 6–6.5) and brittle, with typical feldspar cleavage. It tends to form angular medium-sized particles that support liberation without excessive fines [13]. Rutile (0.8%) also has a Mohs hardness of 6–6.5 and is highly brittle with irregular fractures. It usually forms coarser fragments unless exposed to high comminution energy [32]. Pyrite (0.9%) has a Mohs hardness of 6–6.5 and is highly brittle, shattering along its cubic planes. It forms medium-coarse particles that may require additional grinding to achieve effective liberation [11].

#### 2.2.2. Implications for Liberation Efficiency

Liberation efficiency in mineral processing is closely tied to the nature of particle fragmentation during comminution. Minerals such as quartz and kaolinite tend to disintegrate readily into fine particles, which enhances liberation but also introduces risks associated with overgrinding. The excessive generation of slimes can negatively impact flotation selectivity, while overgrinding of kaolinite, in particular, may lead to increased reagent consumption due to its high adsorption capacity.

In contrast, minerals like Fe-dolomite, albite, and muscovite typically fragment into medium-sized particles. This controlled fragmentation supports a balance between liberation and flotation efficiency, minimizing the production of fines. However, muscovite’s platy morphology can influence pulp rheology, potentially requiring adjustments to flotation parameters to maintain optimal performance. Harder minerals such as pyrite, rutile, and siderite present greater challenges in achieving adequate liberation due to their resistance to breakage. These minerals often require additional grinding energy, and in such cases, HPGR technology may offer advantages. The micro-cracks induced by HPGR can enhance liberation for these tougher phases, improving downstream flotation outcomes without significantly increasing fines.

#### 2.2.3. Optimization of Comminution Strategy

Effective optimization of the comminution strategy hinges on a thorough understanding of differential mineral breakage. Tailoring the approach to the specific breakage behavior of each mineral is essential for maximizing liberation of valuable phases while minimizing the risks of overgrinding. By controlling particle size distribution, flotation efficiency can be significantly improved, ensuring better selectivity and concentrate quality. Moreover, energy consumption can be reduced through the strategic application of targeted comminution techniques. For instance, HPGR-induced micro-cracking is particularly beneficial for hard minerals that resist fragmentation, while VSI is more effective in limiting excessive fines generation, making it suitable for minerals prone to overgrinding. This mineral-specific approach enables a more efficient and sustainable processing workflow.

### 2.3. Methodology

**Figure 2:**
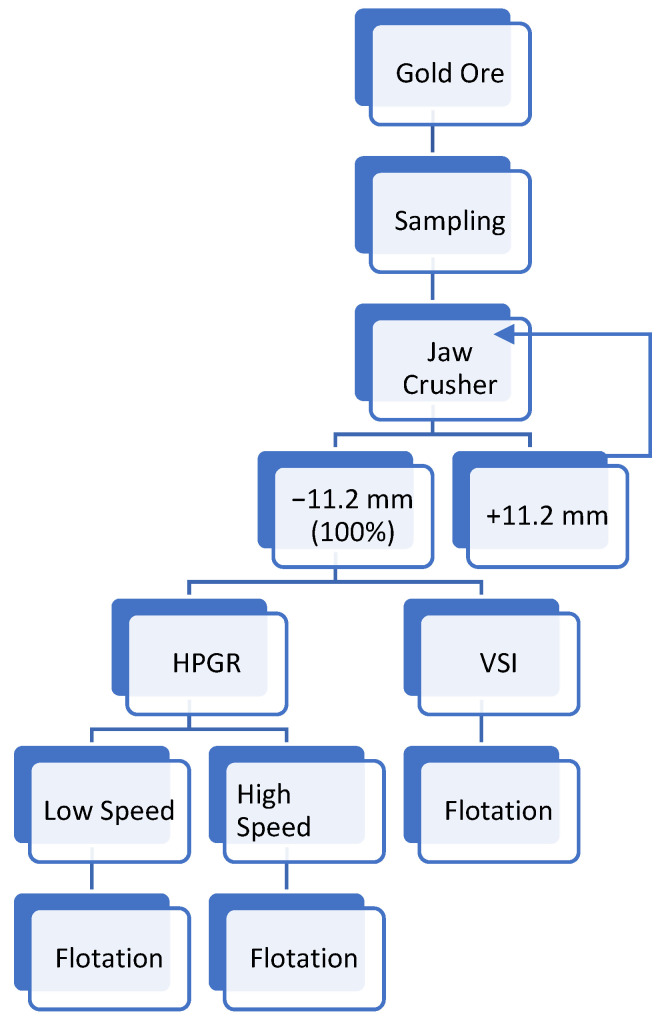
Methodology of the study.

The study scheme is as represented in Figure 2. Cone and quarter sampling is used to obtain 5 kg of gold ore for each experiment. Roughly 4 kg was used for flotation and 500 g for sieve analysis. The ore was crushed with a jaw crusher to −11.2 mm, the required feed size for laboratory-scale VSI and HPGR. PSDS was determined by the wet sieve analysis. VSI and HPGR were used to produce the size fraction + 300 µm to −600 µm. The liberation of the ores in HPGR was optimized by adjusting the pressures in the rolls to obtain the desired product sizes. The flotation and the % gold recovery were determined for each product size from the VSI and HPGR. Efficacies of different collectors were determined on a −300 µm size fraction from VSI and high-speed HPGR. The flotation data were used to optimize the comminution process and the product size range, giving greater flotation and gold recovery. Each test condition was performed without repeats, focusing on a broad comparative framework rather than statistical replication. As such, standard deviations and error bars could not be computed. A rigorous statistical modeling (including uncertainty quantification and predictive confidence) using the same dataset was undertaken in our companion study [27]. This published study applied machine learning and Bayesian techniques to this dataset, providing a robust statistical interpretation of flotation responses under various grinding and reagent conditions.

#### 2.3.1. Experiment Design for HPGR

Experimental conditions for the high-speed HPGR (HS-HPGR) and low-speed HPGR (LS-HPGR) are kept the same. The following parameters were used: feed size (−11.2 mm), sample weight (5 kg), hydraulic pressure (1200 PSI), nitrogen pressure (80, 80 Bar), and power (415 V). The required product sizes are: −600 µm, −425 µm, and −300 µm. The sample was passed number of times to obtain the required particle size. Three passes were required to obtain −600 µm (100%), five passes for −425 µm (100%), and seven passes for −300 µm (100%). The flotation behavior of −300 µm was studied with different collectors to evaluate their influence on gold recovery and grade.

**Figure 3:**
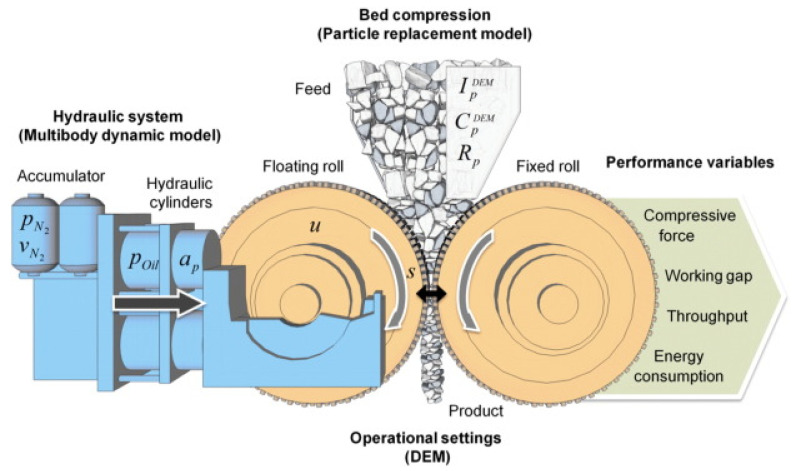
High-Pressure Grinding Rolls.

The material is force-fed into the unit by creating a head of material over the machine, as seen in Figure 3 [13]. Two counter-rotating rolls allow the compression breakage to be used in a continuous rather than batch operation. One of the rollers in the HPGR rotates on a fixed axis while the other is allowed to move linearly with a pressing force applied to the moving roll. The movable roller is forced up against the material in the gap between the rollers by a hydraulic oil cylinder system. This oil pressure acts through cylinders and transmits the grinding force over the cross-section of the diameter of the rolls, where the bed has formed. Since the nitrogen is compressible, if the material is put into the HPGR and the forces push the floating roller back, then the nitrogen will be compressed. By contrast, hydraulic fluid is a nearly incompressible substance. Therefore, if only hydraulic fluid is present in the system (no nitrogen), then the forces will be unable to push the rollers back. The operating procedure specifies a minimum nitrogen pressure, which is crucial [34].

The product fineness is controlled by the grinding force applied to the material bed between the rolls, causing micro-cracks and breakage of the particles. The correlation between particle breakage and the grinding force required needs to be determined for each material [34]. HPGR is accepted as a reliable and cost-effective means of tertiary comminution of “hard rock ores”, and as an alternative for SAG mills. Different speeds (high speed and low speed) on HPGR are obtained by operating the electric induction motors in either 4-pole or 8-pole mode (Appendix A) [34].

#### 2.3.2. Experiment Process (VSI)

The experimental scheme of VSI: A 5 kg ore of −11.2 mm is passed several times through the VSI at 47 Hz, to produce 100%, −600 µm, −425 µm, and −300 µm. Unlike HPGR, VSI-treated samples were not subjected to multiple passes to avoid the risk of losing fine particles during handling, which could bias flotation results. Therefore, the sample passed was sieved once, and only the coarser (plus) size fraction was crushed with VSI, till the required amount of the specific particle size fraction was obtained. Only the coarser (plus) size fraction was crushed with VSI to have a greater proportion of coarser particles. That is, to achieve comparable size fractions, HPGR samples were reprocessed through multiple passes, while VSI samples were processed in a single pass to minimize fines loss. Energy input was monitored and normalized as specific energy (kWh/t) to ensure a fair comparison.

The Vertical Shaft Impactor has three interchangeable chambers: Shoe and Anvil, Rock-on-Anvil, and Rock-on-Rock chambers. Shoe and Anvil are used for large feed, mild to medium-abrasive materials. The Rock-on-Anvil chamber is used for high reduction in medium abrasive materials; the enclosed rotor and anvils combine the grinding action of the rotor with high efficiency reduction of the anvils. The Rock-on-Rock chamber is used for all rock types and the most abrasive materials; the enclosed rotor and rock box configuration causes rock-on-rock crushing, which produces the best-shaped and most consistent material with lower wear cost [35].

**Figure 4:**
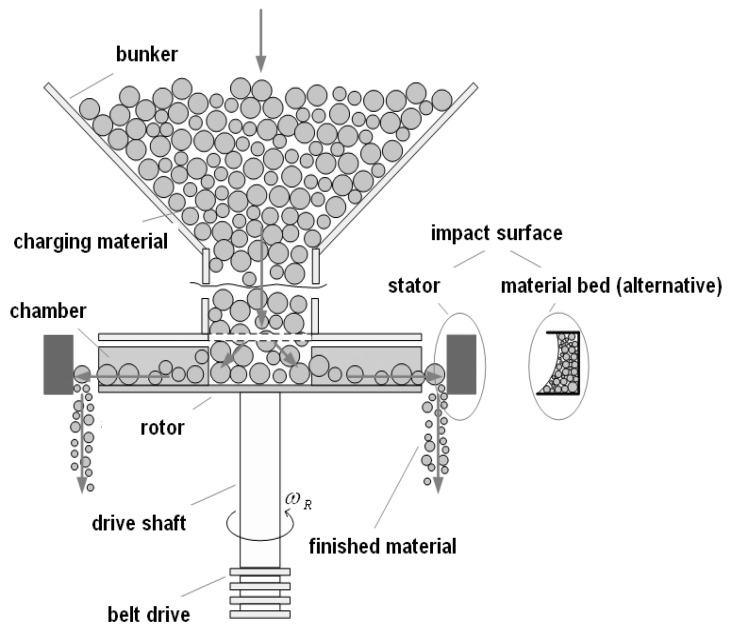
Vertical Shaft Impactor.

**Figure 5:**
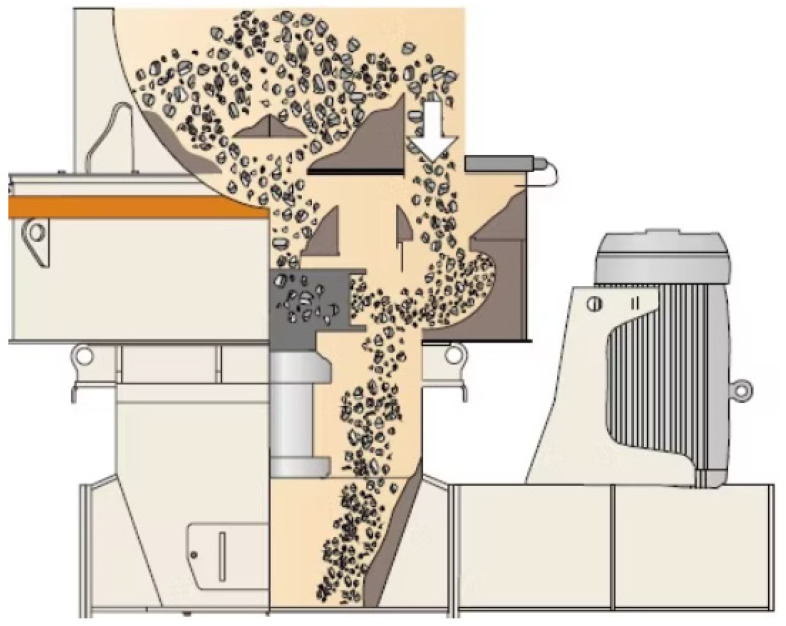
Operating principle of VSI crusher.

Figure 4 illustrates the construction of a VSI. The operating principle of the Vertical Shaft Impact (VSI) crusher is shown in Figure 5. Rock material falls into the rotor, which rotates so that the edge speed is about 50–60 m/s. The rock particles accelerate and impact the surrounding bed of rock that is formed during operation. They also impact rock particles that do not pass through the rotor but fall down outside it. This material is called bi-flow or cascade. Crusher manufacturers recommend operating the VSI crusher with 10–15% of the total as biflow. The fraction of bi-flow is controlled by adjusting hatches in the feed bin above the rotor. The particle breakage in the VSI is achieved through rock against rock impact [9].

#### 2.3.3. Froth Flotation Experiments

To a 30s aerated slurry of ground ore (specific gravity of the ore = 2.8 g/cm^3^), 50 g/t of CuSO_4_ (activator) and 100 g/t of collectors (either PAX (supplied by Lianyungang Huaihua International Trade Co., Ltd., Lianyungang, China), collector-1 or DSP002 (Shark Chemical Global, Johannesburg, South Africa), collector-2) were added, followed by aeration for further 30 s. Finally, 3 drops (17 g/t) of Frother (DSF002A) were added to the slurry. PAX and DSP002 were supplied by Orica Mining Chemicals, (Melbourne, VIC, Australia). Percent solids: 34% solids (3919.12); volume of cell: 9 L; type of cell: Denver flotation cell. The froth was scraped from the cell every 10 s to produce a concentrate of the target mineral. The second collector used for the −300 µm experiments of HS-HPGR and VSI was DSP002 (Di Butyl Dithiophosphate). Each flotation test was performed once due to sample limitations. While replicate testing would have strengthened the analysis, consistent trends across size fractions and collectors support the robustness of the results.

## 3. Results and Discussion

### 3.1. Product Size Distributions (PSDs)

**Figure 6:**
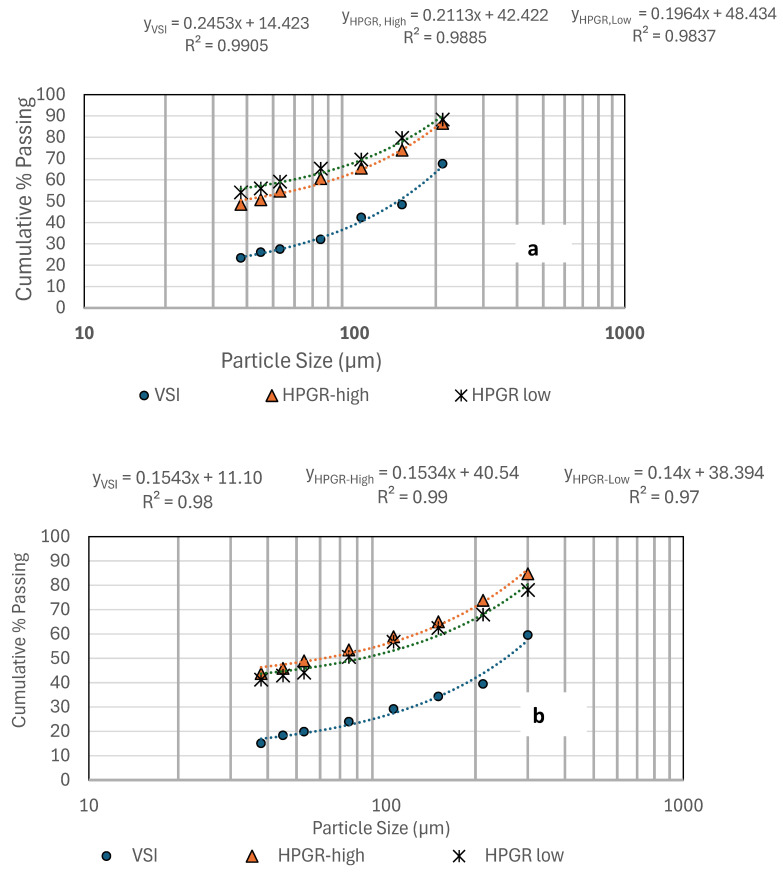

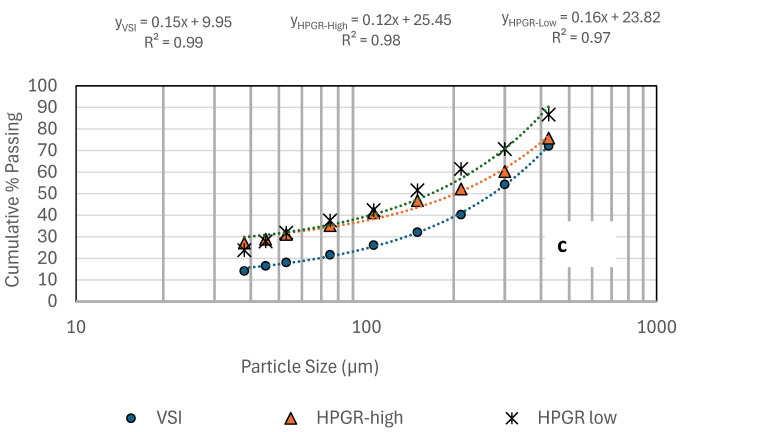
Comparison of size distributions of HPGR products with VSI products. (**a**) −300 µm, (**b**) −425 µm, (**c**) −600 µm.

The PSDSs of the HPGR and VSI products for the sizes −300 µm, −425 µm, and −600 µm are shown in Figure 6a–c. The HPGR produced finer particles for all the sizes with a cumulative % passing of about 45% (−38 µm), whereas the PSDSs of the VSI products are coarser with less cumulative % passing to about 14–20% (−38 µm). For VSI, as the sample did not undergo as many passes, the PSDSs were coarser than the HPGR. Though the PSDSs of LS-HPGR and HS-HPGR showed similar cumulative % passing, the LS-HPGR products were finer than the HS-HPGR products.

### 3.2. Effect of Nitrogen Gas Pressure on HPGR

**Figure 7:**
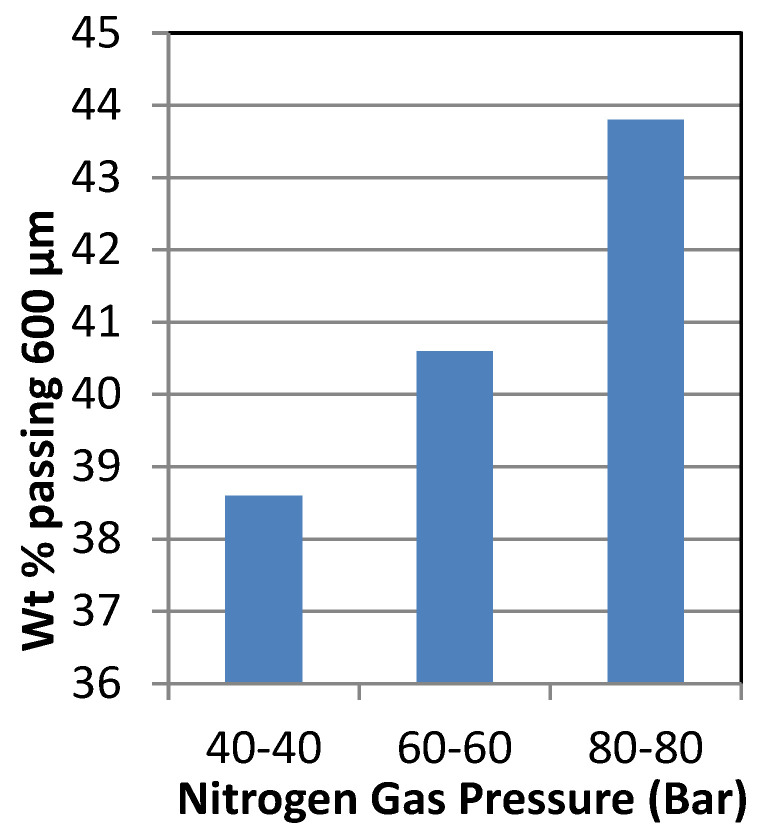
Effect of nitrogen gas pressure on HPGR crushing at 600 µm.

**Figure 8:**
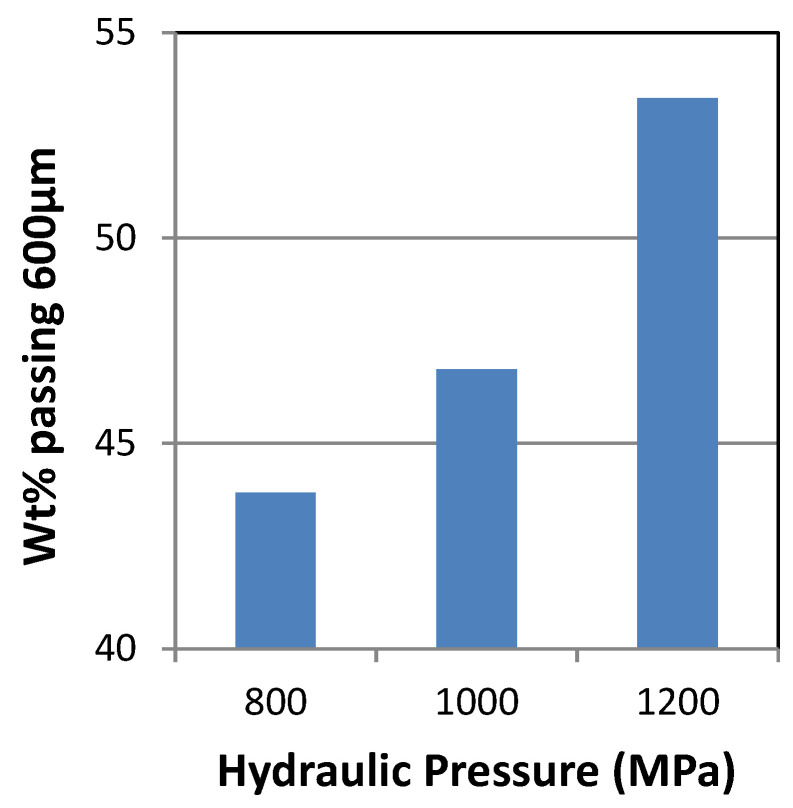
Effect of hydraulic pressure at constant nitrogen gas on HPGR crushing at 600 µm.

Increasing the nitrogen gas pressure increases the wt% passing from 38% to 44% for 600 µm (Figure 7). At (80,80) Bar, the highest wt% passing was 44%. according to Klymowsky et al. 2002 [34] observed a similar phenomenon. Increasing the hydraulic pressure from 800 Psi to 1200 Psi at constant nitrogen pressures of (80,80) Bar increases the wt% passing from 44% to 54% (Figure 8). The highest wt% passing at 54% was at 1200 Psi with (80,80) Bar. As the hydraulic pressure increased, the applied grinding force increased between the rolls, causing micro-cracks and breakage of the particles to produce finer products [34].

### 3.3. Power and Time Required for HPGR Experiments

The power, time, and specific energy required to produce both HS and LS HPGR products are shown in Table 3. Specific energy was calculated using the formula:

Specific Energy (kWh/t) = (Power × Time)/(Mass in tonnes × 3600)

These values reflect the true energy consumption under controlled test conditions. Values demonstrate a clear increase in energy demand as particle size is reduced under identical pressure and mass conditions. More power is required for LS-HPGR compared to the HS-HPGR. For smaller particle sizes, the power and time increase for both HS-HPGR and LS-HPGR tests, and the specific energy for HS-HPGR and LS-HPGR increases for smaller sizes.

### 3.4. Effect of Particle Size Versus Speed Versus Power Consumption

**Figure 9:**
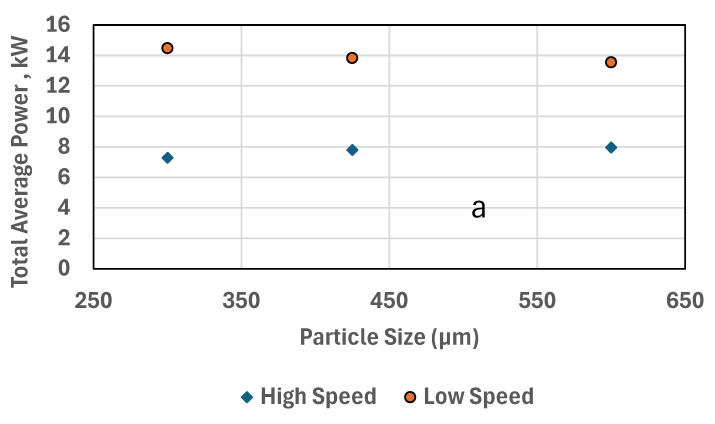

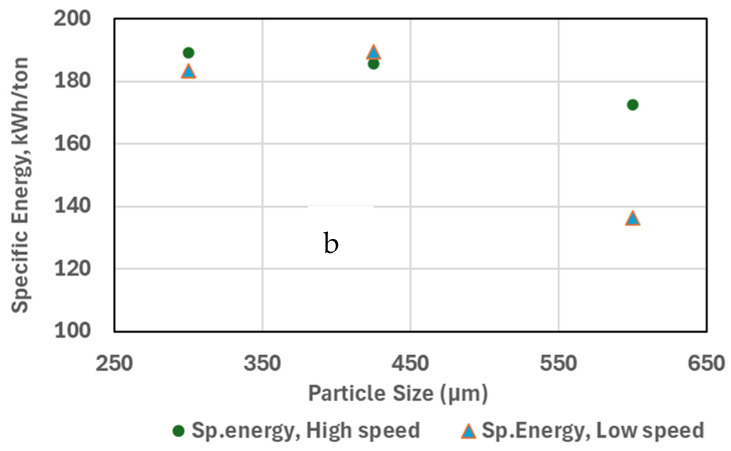
The effect of (**a**) particle size for various speeds versus power consumption, (**b**) particle size for various speeds versus power consumption.

Power consumption and specific energy for low and high speeds HPGR producing different PSDS are illustrated in Figure 9a,b. The maximum power consumption of low speed at about 14.5 KW is approximately twice that of high speed-HPGR at 8 KW. The power for high-speed HPGR decreases with decreasing particle size. The specific energy increases for both high and low speeds for smaller sizes. Power and specific energy can behave differently due to their distinct relationships with throughput and operational conditions: Power increases for low-speed HPGR because more energy is needed to compress and crush material at lower speeds, where the compression force is higher and the processing time is longer. In contrast, power decreases for high-speed HPGR due to its more efficient operation, with faster throughput reducing the time and energy required per unit of material processed. Specific energy increases for both high-speed and low-speed HPGR, as finer material demands more energy to break down. For high-speed HPGR, the energy is distributed over a larger volume of material due to high throughput, while for low-speed HPGR, the prolonged processing time and greater force result in higher energy consumption per ton of material.

In summary, power changes with speed due to operational efficiency, while specific energy increases with finer particle sizes due to the increased energy needed to achieve the desired size reduction. For HS-HPGR, the power typically decreases with decreasing particle size because smaller particles are more easily compressed and fractured. As the particle size decreases, the material may exhibit better flow and less resistance, leading to reduced power requirements. Additionally, HS-HPGRs operate at higher velocities, which can enhance the efficiency of size reduction as the particles are subjected to more frequent compressive forces. In contrast, LS-HPGRs operate at lower velocities and might have a different interaction with the material. As particle size decreases, the material can become more resistant to compression and requires more energy to achieve the desired size reduction. Smaller particles may lead to increased friction and higher power consumption, as the HPGR needs to apply more force to process the material effectively.

### 3.5. Flotation Gold Grade Recovery

The comparative results of flotation recoveries of LS-HPGR, HS-HPGR, and VSI using PAX on the −300 µm, −425 µm, and −600 µm are summarized in Table 4.

#### 3.5.1. −300 µm Experiments

**Figure 10:**
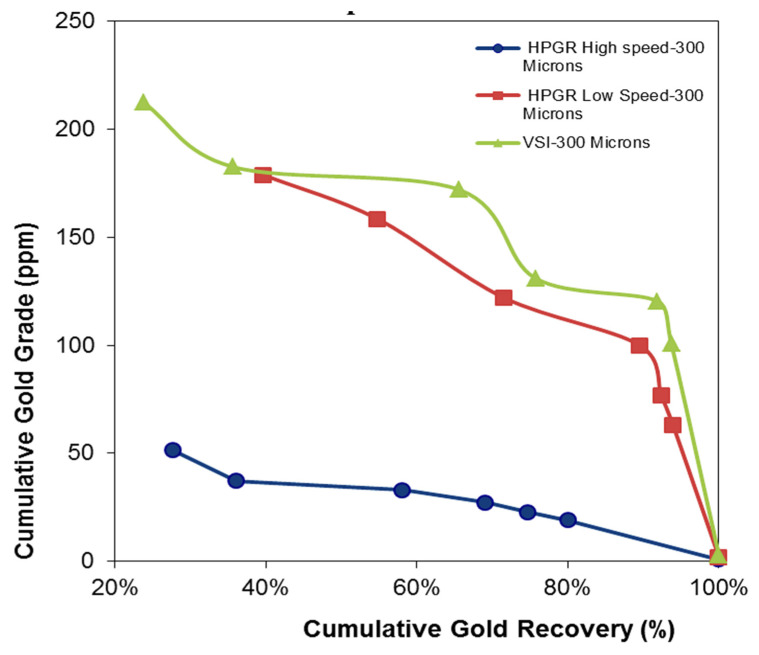
Illustrating gold grade recovery curve for −300 µm experiments.

Gold grade versus gold recovery curves for −300 µm are presented in Figure 10. The HS-HPGR shows lower cumulative gold grade and recovery, perhaps due to the very low head grade. Gold recovery for VSI is higher than for LS- and HS-HPGR. This could be due to the higher head grade from the sampling effect. As shown in Figure 6a–c, the VSI produces coarser results compared to the HPGR. The high amount of fines and low head grade associated with HS-HPGR have a negative effect on flotation. The fines have less momentum in an agitated slurry, reducing the probability of collision and particle-air bubble attachment, leading to lower gold recovery [22,36].

#### 3.5.2. −425 µm Experiments

**Figure 11:**
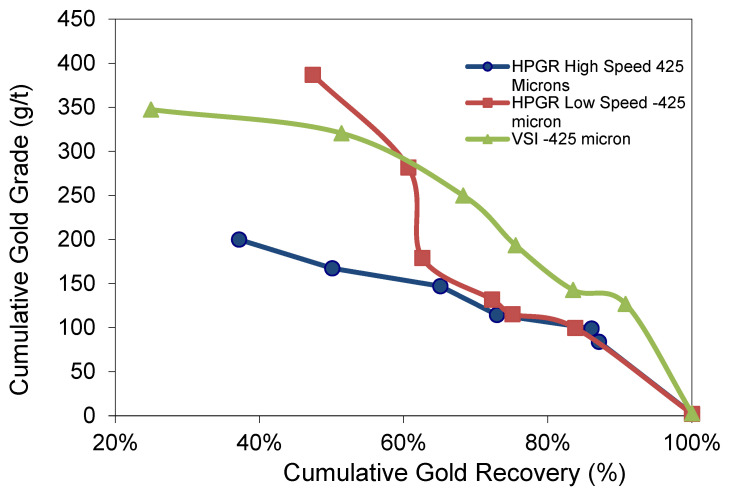
Illustrating gold grade recovery curve for −425 µm experiments.

The LS-HPGR for −425 µm shows 83.8% gold recovery and 99.7 g/t Au gold grade (Figure 11). The HS-HPGR shows a better gold recovery of 87.09% with a lower gold grade of 83.99 g/t Au. The VSI test shows better cumulative gold recovery of about 90.78%, and a cumulative concentrate grade of 126.91 g/t Au. The VSI showing higher recovery and cumulative gold grade than the HPGR is attributed to a greater proportion of coarse particles in the VSI than the HPGR tests.

#### 3.5.3. −600 µm Flotation Experiments

**Figure 12:**
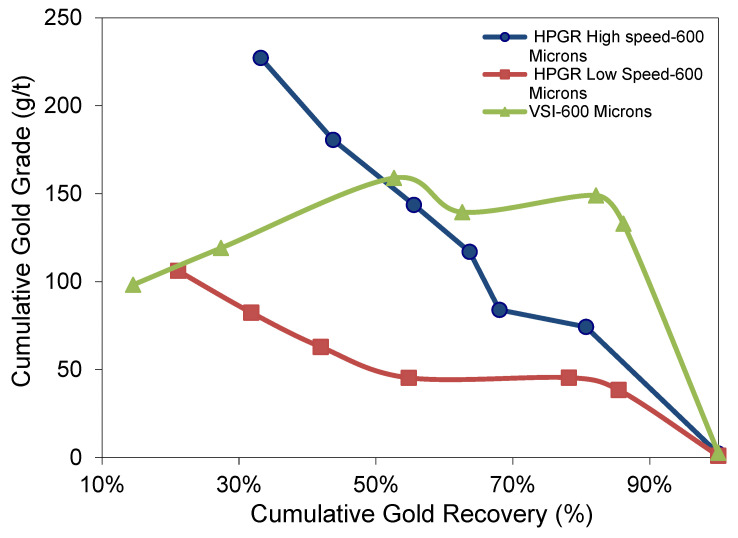
Illustrating gold grade recovery curve for −600 µm experiments.

The flotation gold grade recovery curves for −600 µm using the HS HPGR, LS HPGR, and VSI tests are shown in Figure 12. The LS HPGR showed 85.45% cumulative gold recovery and a gold grade of 38.51 g/t Au. The HS HPGR test shows less gold recovery of 80.67% with a higher concentrate gold grade of 74.32 g/t Au. This is attributed to the low head grade of the LS HPGR test compared to the high head grade for HS HPGR. The VSI test shows better cumulative gold recovery and a cumulative gold grade of 132.92 g/t Au, attributed to the higher head grade/coarser particle size.

**Figure 13:**
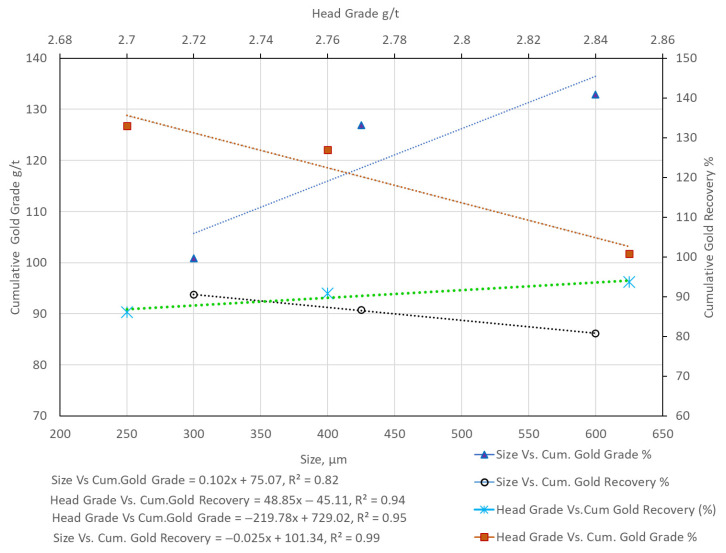
Size vs. cumulative gold recovery and cumulative gold grade; head grade vs. cumulative gold recovery and cumulative gold grade for VSI products.

**Figure 14:**
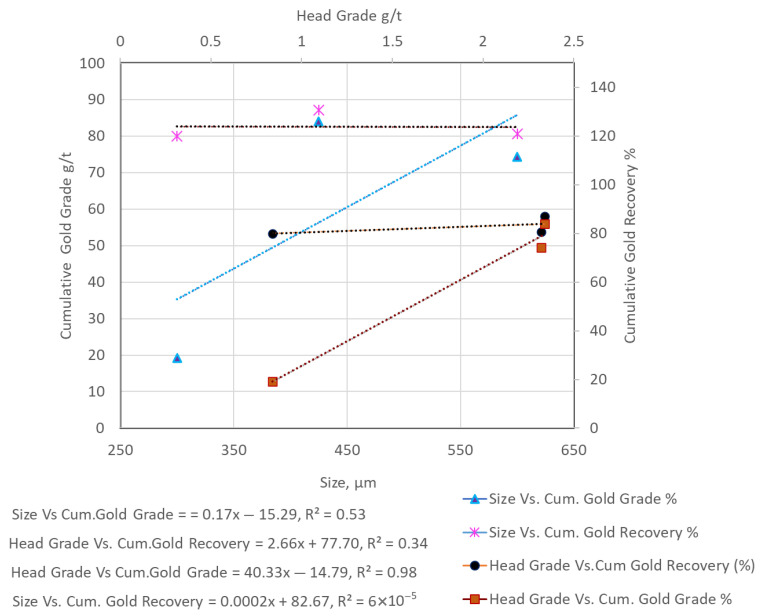
Size vs. cumulative gold recovery and cumulative gold grade; head grade vs. cumulative gold recovery and cumulative gold grade for HS-HPGR products.

**Figure 15:**
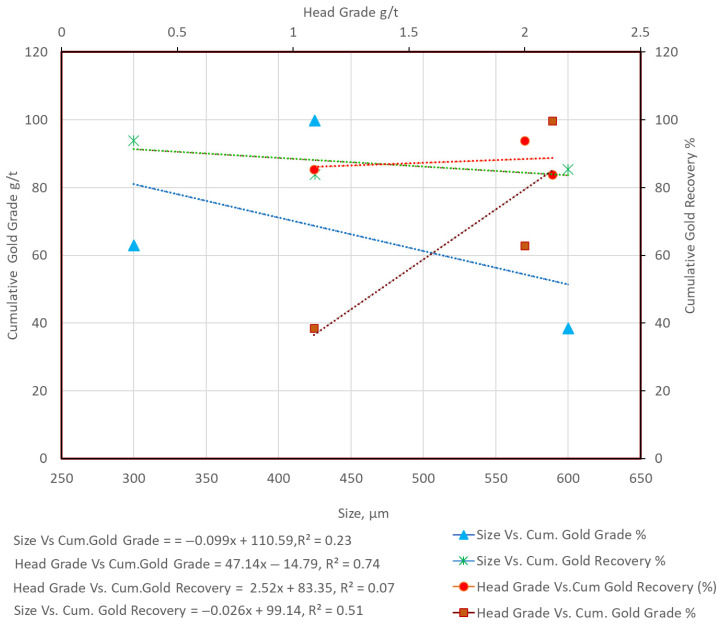
Size vs. cumulative gold recovery and cumulative gold grade; head grade vs. cumulative gold recovery and cumulative gold grade for LS-HPGR products.

When comparing flotation results, variations in head grade must be carefully considered. For example, the relatively low flotation grade and recovery for HS-HPGR at −300 µm (Table 4) are partly attributable to a low head grade of just 0.84 g/t Au, compared to 2.85 g/t for VSI. In our results, higher head grades often correlated with better flotation outcomes. To clarify this, Figure 13, Figure 14 and Figure 15 compare head grade against cumulative recovery and grade for each comminution mode. These visualizations reveal that while the comminution method plays a dominant role, head grade introduces a secondary influence on flotation performance. Therefore, while the flotation trends across VSI and HPGR are primarily attributable to differences in particle size and liberation, variations in head grade may account for some of the observed fluctuations in performance. This limitation is inherent to single-pass, non-replicated test designs and is acknowledged in our interpretation.

In an attempt to isolate the effects of size and head grade on the gold recovery and the gold grade for all the size fractions (−300 µm, −425 µm, and −600 µm) from VSI, HS-HPGR, and LS-HPGR, Figure 13, Figure 14 and Figure 15 are presented.

Figure 13 shows opposing trends between size vs. cumulative gold recovery (decreasing, slope = −0.025) and head grade vs. cumulative gold recovery (increasing, slope = 48.85). Similarly, size vs. cumulative gold grade increases (slope = 0.102) and head grade vs. cumulative gold grade decreases (slope = −219) for VSI products. This phenomenon is similar to the observation made by [20], who reported that the gold recovery increased with fine size fractions. However, the gold grades in fines fractions were relatively low compared to those of coarse size fractions (Table 4). Figure 14 shows similar trends between size vs. cumulative gold recovery (flat trend) and head grade vs. cumulative gold recovery (flat trend) for the HS-HPGR products. Head grade vs. cumulative recovery has a slightly higher slope value. For the size vs. cumulative recovery slope value, the effect is not pronounced. Size vs. cumulative gold grade shows an increasing trend, and also an increasing trend for head grade vs. cumulative gold grade. Figure 15 shows the almost similar trends between size vs. cumulative gold recovery (slightly decreasing trend, negative slope) and head grade vs. cumulative gold recovery (a flat trend, but slightly positive slope). Opposite trends were observed for size vs. cumulative gold grade (increase and then decreasing) and head grade vs. cumulative gold grade (increasing) trends for the LS-HPGR products.

#### 3.5.4. Recovery vs. Grade at Coarser Sizes

At larger particle sizes, lower overall surface area leads to fewer bubble-particle collisions, which can reduce recovery. However, coarser particles are more likely to be fully liberated and less prone to slime coating, leading to higher concentrate purity (grade). This explains the inverse relationship between recovery and grade as particle size increases.

In summary, flotation performance varied significantly across comminution methods and particle sizes. VSI consistently produced higher flotation efficiencies across all size fractions, especially at 600 µm, where recovery and grade were both maximized. HPGR, particularly in high-speed mode, generated excessive fines that increased recovery but reduced grade due to entrainment. These results demonstrate that particle morphology and energy input strongly influence flotation selectivity and overall efficiency.

### 3.6. Influence of Collectors on Flotation (PAX Versus Dithiophosphate)

**Figure 16:**
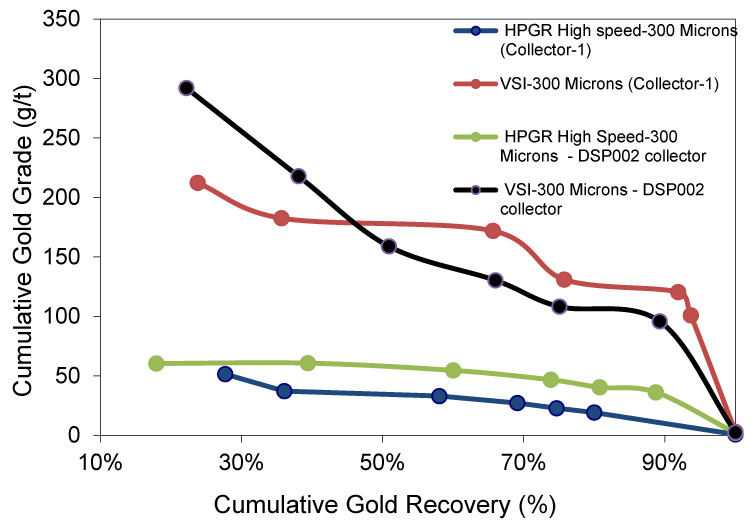
Influence of collectors on flotation gold grade and recovery for 300 µm HS HPGR and 300 µm VSI.

Collectors’ influence on gold flotation recovery for −300 µm from HS HPGR and VSI is shown in Figure 16 and Table 5. The PAX (Potassium Amyl Xanthate), Collector 1, shows less gold recovery of 79.98%, for the HS-HPGR product with a low concentrate grade (19.15 g/t Au). This may be in part due to the low head grade of about 0.84 g/t Au. Collector 2, DSP002 (Di Butyl Dithiophosphate C_8_H_18_NaPS_2_), for the HS HPGR product gives a higher gold recovery of 89.72%, and a higher concentrate gold grade of 36.17 g/t Au. PAX appears to perform better on coarse (VSI −300 µm) particles compared to the fines (HP-HPGR, −300 µm) and vice versa, though the effect of low head grade for HP-HPGR (−300 µm) cannot be neglected. It should be noted that PAX with C = 6 can render the gold particles less hydrophobic than the DSP002 with C = 8. The collector DSP002 is a specific fine gold collector [22], which renders the fine gold particles more hydrophobic (due to high surface area and surface energy facilitating better attachment to the air bubbles) than the bulk collector PAX, which stabilizes the froth better to help float the coarse particles. The DSP002 makes the gold fines more water-repellent than the PAX and helps with stronger particle collision and attachment with air bubbles than the PAX [22], resulting in higher gold recovery with a higher concentrate grade.

Overall, DSP002 showed better performance with HS-HPGR-treated fine particles due to enhanced adsorption on micro-cracked surfaces, while PAX was more effective with VSI-generated particles due to its affinity for coarse, clean surfaces. These trends confirm the influence of particle characteristics on collector efficacy and highlight the importance of matching collector chemistry to the comminution product for optimized flotation outcomes.

### 3.7. Indirect Evaluation of Liberation from Flotation Performance

**Figure 17:**
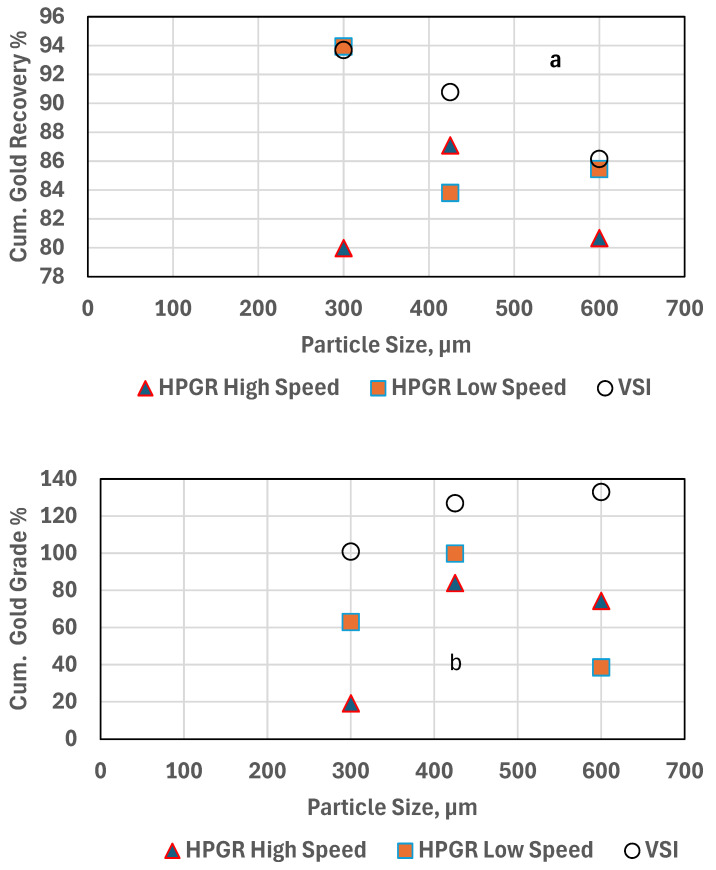
(**a**) Size versus cumulative gold recovery; (**b**) size versus cumulative gold grade.

Though direct evaluation of gold dissociation or liberation was not a primary objective of this study, its significance in the flotation of coarse-grained gold ore is acknowledged. The flotation results offer indirect evidence of gold liberation. The recovery and grade of gold in the flotation concentrates serve as indicators, with higher values suggesting improved liberation of gold particles, as illustrated in Figure 15. Additionally, the size-by-size analysis of flotation outcomes reveals how gold recovery and grade vary with particle size, providing further insight into liberation behavior. Differences in flotation performance between HPGR and VSI crushed samples also point to variations in liberation efficiency. Moreover, the response to different flotation reagents, as shown in Table 5 and Figure 16, indirectly reflects the accessibility of gold surfaces, which is closely related to liberation. The trend of increasing cumulative gold recovery alongside decreasing cumulative gold grade with finer particle sizes, as depicted in Figure 17, offers valuable insights into the mineral liberation process.

Increasing Cumulative Gold Recovery

This indicates that as particle size decreases, more gold is being recovered in the process. This is often a sign that finer particles contain more liberated gold, making it easier for the recovery process to extract it.

Decreasing Cumulative Gold Grade

A decrease in gold grade with decreasing particle size suggests that while more gold is being recovered, the proportion of gold in the recovered material is lower. This could mean that as the particles get finer, more gangue (non-valuable) material is also being recovered along with the gold.

Implications for Liberation

Liberation: This pattern suggests that finer grinding is leading to better liberation of gold, meaning that more gold particles are being freed from the surrounding ore. However, it also indicates that finer particles may still contain some attached gangue material, which dilutes the grade. Trade-off: The decreasing grade with increased recovery reflects a trade-off where finer grinding liberates more gold but also increases the recovery of non-valuable material. In summary, the observed trend indicates that finer grinding improves liberation, leading to higher gold recovery, but it also brings along more gangue, reducing the overall grade of the recovered material.

These observations reinforce the classic trade-off between liberation and selectivity. HPGR improves mineral liberation through micro-cracking, but this benefit is offset by overgrinding and increased slime generation, which reduce flotation selectivity. VSI, in contrast, produces coarser, cubical particles with fewer fines, yielding higher-grade concentrates and better flotation efficiency at lower energy input. This underscores the need for careful balancing of grinding intensity with flotation circuit requirements.

#### 3.7.1. Flotation Efficiency

The flotation efficiency, ƞ, is calculated using the formula:

$$\eta =\frac{Cumulative\;Gold\;recovery\;\times Cumulative\;grade\;}{Head\;grade}$$

The results are listed in Table 4. The calculated flotation efficiencies range from 1823 to 4241, which aligns well with expected industry values for gold flotation. Here is a breakdown of how these values compare with domain knowledge and what they suggest about comminution effects on flotation performance.

#### 3.7.2. Key Observations and Interpretation

This study highlights the superior performance of Vertical Shaft Impactor (VSI) technology over High Pressure Grinding Rolls (HPGR) in achieving high flotation efficiency across various particle sizes. VSI at 600 µm delivers the highest flotation efficiency (4241), followed closely by VSI at 425 µm (4174) and 300 µm (3315). These results affirm VSI’s effectiveness in producing well-liberated, cubical particles that enhance flotation selectivity and concentrate grade. The coarser yet more uniform particle distribution generated by VSI appears to minimize the formation of slimes, thereby improving attachment to air bubbles and reducing fines-related losses. This supports VSI’s suitability for coarse flotation applications.

HPGR, while generally less efficient than VSI, shows its best performance at the intermediate grind size of 425 µm. LS-HPGR and HS-HPGR at this size achieve flotation efficiencies of 3943 and 3126, respectively. This suggests that HPGR’s ability to induce micro-cracks enhances gold recovery, but its tendency to generate excessive fines can compromise selectivity and grade. At 600 µm, HPGR performance declines (LS-HPGR: 3019; HS-HPGR: 2584), and at 300 µm, the impact of overgrinding becomes more pronounced. HS-HPGR at this size records the lowest flotation efficiency (1823), likely due to excessive fines production, which leads to entrainment losses, increased reagent consumption, and reduced concentrate quality.

Overall, VSI consistently outperforms HPGR in flotation efficiency, particularly at coarser and intermediate sizes. HPGR shows promise at 425 µm, where it balances liberation and selectivity, but its performance diminishes at finer sizes due to overgrinding. Flotation efficiency trends are shown in Table 6.

## 4. Discussion

The comminution process significantly influences flotation performance by altering particle size distribution, shape, surface chemistry, and liberation efficiency [37,38]. VSI and HPGR operate under fundamentally different breakage mechanisms, affecting downstream processing in distinct ways.

### 4.1. Vertical Shaft Impactor (VSI)—Impact and Attrition Breakage

Vertical Shaft Impactors (VSI) predominantly rely on high-velocity impact and attrition to break particles along their natural fault lines [32]. This mode of comminution produces a product that is typically more cubical and uniformly shaped, characteristics that enhance flotation selectivity by facilitating improved particle–bubble attachment. Furthermore, VSI tends to limit excessive fines generation, thereby reducing slime formation. This is beneficial for flotation as it lowers reagent consumption and minimizes entrainment losses associated with ultrafine particles.

### 4.2. High-Pressure Grinding Rolls (HPGR)—Inter-Particle Compression and Micro-Crack Formation

High-Pressure Grinding Rolls (HPGR) operate by applying inter-particle compression, which results in the formation of micro-cracks that enhance mineral liberation. These micro-cracks increase the available surface area for collector adsorption, potentially improving flotation response. However, the same mechanism may also lead to overgrinding in finer size fractions, generating excessive slimes that can impair flotation by increasing entrainment and reducing selectivity. The degree of micro-cracking and its impact on flotation performance are influenced by operational parameters such as applied pressure, roll speed, and feed moisture content.

### 4.3. Flotation Performance: Surface Chemistry and Recovery Trends

The flotation performance of VSI- and HPGR-crushed particles can be attributed to differences in surface properties, hydrophobicity, and particle size effects [39]. Particles generated by HPGR typically exhibit higher surface roughness due to micro-crack formation, which can enhance collector adsorption and improve recovery, especially for finely disseminated gold [40]. However, this increased roughness may also promote bubble–particle detachment, particularly in ultra-fine size fractions where stable attachment is more difficult to maintain. In contrast, particles produced by VSI crushing are generally smoother and more uniformly shaped, allowing for the formation of more stable bubble–particle aggregates. This contributes to improved flotation selectivity and higher concentrate grades.

### 4.4. Particle Size Effects and Flotation Selectivity

Flotation recovery generally improves at moderate particle sizes (425–600 µm), where sufficient liberation is achieved without generating excessive slimes. At these sizes, the balance between particle morphology and surface chemistry favors effective bubble–particle attachment. The lowest flotation efficiency was observed in HS-HPGR at 300 µm, indicating that overgrinding leads to excessive fines generation, which reduces flotation selectivity and increases entrainment losses. Conversely, VSI at 600 µm achieved the highest flotation efficiency (4241.13) at a specific energy of just 9.79 kWh/t, highlighting the advantage of coarse, well-liberated particles produced at low energy input.

**Figure 18:**
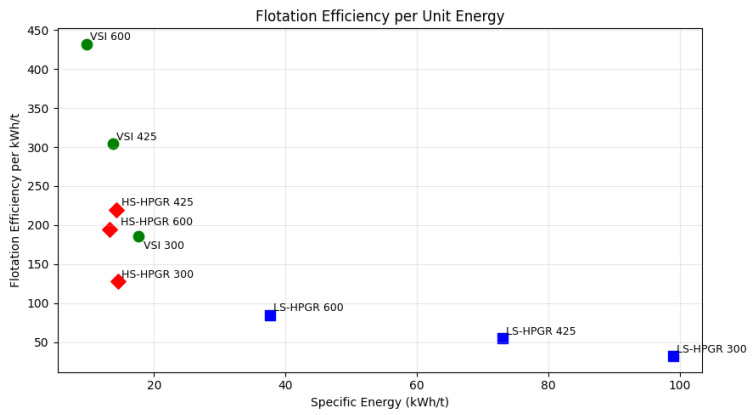
Flotation efficiency per unit of specific energy input (flotation efficiency ÷ kWh/t) for each comminution mode and size fraction.

A broader evaluation of energy input and flotation performance reveals that although high flotation efficiency can also be achieved at higher energy inputs (e.g., LS-HPGR 425 at 73.04 kWh/t yielding 3943.74 efficiency), the efficiency per unit energy input is substantially lower (Figure 18). In contrast, VSI consistently achieved high flotation efficiency with much lower energy demand. For instance, VSI 425 delivered an efficiency of 4174.24 at only 13.80 kWh/t. This demonstrates that while some HPGR configurations can reach competitive flotation performance, VSI offers a significantly better energy-to-efficiency ratio, indicating a more sustainable comminution–flotation pathway.

Overall, there is no fixed threshold for optimal flotation efficiency; instead, the data support a strategy of maximizing flotation output per unit of energy. This aligns with sustainability objectives and emphasizes the importance of energy-efficient comminution in modern flotation circuits. Notably, factors such as particle shape, surface properties, and collector chemistry also contribute to performance, as seen with the VSI 300 µm test, which outperformed HS-HPGR 300 µm despite a slightly higher energy input.

### 4.5. Scale-Up Considerations

While the present study provides a laboratory-scale comparison of VSI and HPGR comminution strategies, several considerations must be made when translating these findings to industrial scale. First, slurry rheology in full-scale flotation cells can significantly impact bubble-particle collision efficiency and froth stability. VSI products, which contain a lower proportion of fines and more uniform coarse particles, may facilitate improved slurry flow and reduce viscosity, potentially enhancing scale-up flotation performance.

Second, wear rates and maintenance cycles differ between HPGR and VSI in industrial settings. HPGR units tend to experience increased roller wear with harder ores and may require frequent hydraulic pressure adjustments. VSI units, although energy-efficient, may experience wear depending on chamber configuration (e.g., rock-on-rock vs. anvil designs) [35]. Third, flotation kinetics and residence time in large-scale tanks differ from lab-scale cells. The interaction of coarse particles generated from VSI with industrial impellers and air dispersion systems needs further investigation. Coarse particles may settle or bypass froth recovery in conventional cells, which necessitates further pilot-scale tests or deployment in coarse flotation circuits (e.g., Eriez HydroFloat) [41]. Finally, collector dosage and froth recovery strategies must be scaled in proportion to ore tonnage and surface area distribution. As our study indicates, VSI-generated particles tend to require lower reagent input due to reduced surface area per unit mass—this may lead to cost savings and more sustainable operations at scale.

### 4.6. Collector Mechanisms and Particle Chemistry

The observed differences in flotation recovery between PAX and DSP002 across the tested comminution products are closely linked to particle size and surface properties. PAX (Potassium Amyl Xanthate), with a shorter hydrocarbon chain (C5–C6), is known to be effective on coarse and moderately liberated sulfide particles. In contrast, DSP002 (Di Butyl Dithiophosphate) contains longer alkyl chains (C8) and a phosphorodithioate group, enhancing its selectivity for fine or poorly liberated sulfidic gold. The higher performance of DSP002 on HS-HPGR-derived fines can be attributed to its stronger chemisorption onto mineral surfaces with high surface energy and micro-cracks—enhancing the hydrophobicity of gold fines and promoting bubble attachment. Conversely, PAX stabilizes the froth better and shows improved performance with coarser VSI particles due to its ability to maintain larger bubble-particle aggregates in a less turbulent flotation environment.

While zeta potential measurements were not directly obtained in this study, it is known that xanthates and dithiophosphates impart different electrokinetic shifts on sulfide surfaces, which influence adsorption and subsequent flotation. Prior studies confirm that dithiophosphates perform better in systems with high fines and lower pH sensitivity. These known mechanisms align with our experimental findings, providing a plausible explanation for the collector-specific responses observed.

### 4.7. Environmental and Sustainability Considerations

The flotation circuit’s sustainability is influenced not only by energy efficiency but also by reagent use and environmental footprint. In our study, VSI-crushed samples produced coarser particles with reduced slime generation compared to HPGR, particularly in high-speed mode. Since fines and slimes tend to increase collector and frother consumption due to their higher surface area and adsorptive capacity, VSI’s coarse product may offer a more environmentally benign profile by reducing reagent demand. Additionally, overgrinding in HPGR led to excessive fines, which in industrial practice can cause elevated tailings treatment costs, higher water demand, and increased reagent residues in process water. The ability of VSI to achieve high flotation efficiency at lower specific energy and reduced fines suggests not only operational cost savings but also lower indirect emissions and reduced chemical footprint. Future life cycle analysis (LCA) could quantify these benefits in terms of greenhouse gas reduction, reagent toxicity, and water use, supporting broader sustainability goals in mineral processing.

## 5. Conclusions

This study demonstrates that grinding mechanisms significantly influence flotation performance, with High-Pressure Grinding Rolls (HPGR) enhancing mineral liberation through micro-crack formation, while Vertical Shaft Impactors (VSI) offer superior flotation selectivity due to their ability to produce coarser, more uniformly shaped particles. By quantifying flotation efficiency and linking it to specific energy consumption and particle characteristics, this work provides a rigorous framework for optimizing gold flotation circuits.

Key findings reveal that VSI, with minimal passes, consistently produced coarser particles that achieved higher gold recovery and grade across all tested size fractions. In contrast, HPGR required multiple passes to achieve similar size reductions, resulting in finer particles that negatively impacted flotation performance due to excessive slime generation. The use of tailored collectors further highlighted that PAX was more effective for coarser VSI products, while DSP002 improved recovery in finer HPGR fractions, particularly below 300 µm.

Power consumption analysis showed that high-speed HPGR benefited from operational efficiency, with decreasing power requirements at finer sizes. However, low-speed HPGR exhibited increased power demand due to prolonged compression and higher resistance. Specific energy increased for both HPGR modes as particle size decreased, underscoring the energy cost of fine grinding.

Importantly, flotation efficiency was highest for VSI at 600 µm (4241) with a specific energy of just 9.79 kWh/t, while the lowest efficiency was observed in HS-HPGR at 300 µm (1823), despite a lower energy input. These results confirm that overgrinding not only increases energy consumption but also reduces flotation selectivity and concentrate quality.

In conclusion, this study underscores the critical role of particle size management, comminution strategy, and collector selection in optimizing gold recovery. The findings support the integration of VSI in energy-conscious flotation circuits and suggest that hybrid approaches—combining HPGR for liberation and VSI for particle shaping—may offer a balanced path toward maximizing recovery while minimizing energy use. These insights provide a valuable foundation for future research and industrial implementation in sustainable mineral processing.

These findings also highlight a clear liberation–selectivity trade-off. HPGR promotes mineral liberation via micro-cracking, which enhances flotation recovery but can lead to excessive fines and lower selectivity at finer sizes, particularly at −300 µm. In contrast, VSI produces coarser, cubical particles with reduced slimes, supporting higher flotation grades and improved selectivity, especially at lower energy input. Although direct mineral liberation quantification (e.g., via QEMSCAN or MLA) was not conducted, trends in flotation efficiency, size-specific recovery/grade, and collector performance collectively provide indirect evidence of this trade-off. Future studies incorporating quantitative liberation analysis are recommended to validate and deepen these insights for circuit optimization.

## Figures and Tables

**Table 1:** Gold recovery with respect to size [20].

Size (μm)	Gold Recovery (%)	Sulfur Recovery (%)	Gold Grade (%)
38–53	92.4	89.1	36.6
53–75	88.5	86.8	43.9
75–106	90.0	87.0	67.6
106–150	87.7	80.0	83.4
150–212	90.0	>80.0	127.4
212–300	76.2	66.7	58.5

**Table 2:** Ore composition wt%.

Phase	Weight%
Quartz	50
Muscovite	18
Fe-Dolomite/ankerite	11.5
Chlorite	7.9
Siderite	4.2
Kaolinite	3.4
Albite	3.3
Rutile	0.8
Pyrite	0.9

**Table 3:** Power consumption and specific energy for HPGR and VSI tests.

Test	Power (kW)	Time (s)	Sample Mass (kg)	Specific Energy (kWh/t)
HS-HPGR 600	7.97	30	5	13.28
LS-HPGR 600	13.55	41.5	5	37.65
VSI 600	5.42	32.5	5	9.79
HS-HPGR 425	7.8	33	5	14.3
LS-HPGR 425	13.83	95	5	73.04
VSI 425	5.52	45	5	13.8
HS-HPGR 300	7.29	36	5	14.58
LS-HPGR 300	14.48	122	5	98.94
VSI 300	5.78	55	5	17.66

**Table 4:** Flotation results for PAX.

Crusher/Size µm	Cumulative Gold Recovery (%)	Cumulative Gold Grade (g/t)	Head Grade, (g/t)	Flotation Efficiency
HS-HPGR (600 µm)	80.67	74.32	2.32	2584.22
LS-HPGR (600 µm)	85.45	38.51	1.09	3018.97
VSI (600 µm)	86.15	132.92	2.7	4241.13
HS-HPGR (425 µm)	87.09	83.99	2.34	3125.94
LS-HPGR (425 µm)	83.8	99.77	2.12	3943.74
VSI (425 µm)	90.78	126.91	2.76	4174.24
HS-HPGR (300 µm)	79.98	19.15	0.84	1823.35
LS-HPGR (300 µm)	93.93	62.9	2	2954.10
VSI (300 µm)	93.69	100.85	2.85	3315.31

**Table 5:** PAX vs. DSP002 flotation results.

Test	PAX			Di Butyl Dithiophosphate, DSP002		
	Cumulative Gold Recovery (%)	Cumulative Gold Grade (g/t)	Head Grade (g/t)	Cumulative Gold Recovery (%)	Cumulative Gold Grade (g/t)	Head Grade (g/t)
HS-HPGR (300 µm)	79.98	19.15	0.84	88.72	36.17	2.02
VSI (300 µm)	93.93	100.85	2.85	89.26	95.80	2.46

**Table 6:** Flotation efficiency trends.

Expected Trend	Observed in Data?	Explanation
HPGR improves liberation, but can reduce selectivity if too fine	Yes	LS-HPGR (600 and 425 µm) shows improved efficiency, but 300 µm struggles
VSI produces coarser, well-liberated particles with better flotation selectivity	Yes	VSI has the highest flotation efficiency across all sizes
Overgrinding (e.g., 300 µm) leads to slimes and flotation inefficiency	Yes	HS-HPGR (300 µm) has the lowest flotation efficiency

## Data Availability

The original contributions presented in this study are included in the article. Further inquiries can be directed to the corresponding author.

## Appendix A

HPGR motor speeds (4-pole/8-pole) and specific energy calculations:

Different operating speeds of the HPGR (high-speed and low-speed modes) were achieved by using electric induction motors configured as either 4-pole or 8-pole systems. For a 50 Hz power supply, typical in Australia, the synchronous speed of a 4-pole motor is 1500 rpm, and for an 8-pole motor, it is 750 rpm. Based on an estimated roll diameter of 0.5 m, the corresponding tip speeds were approximately 39.3 m/s (4-pole) and 19.6 m/s (8-pole), respectively. These differences influence the contact time and energy applied during inter-particle compression. A control panel was used to toggle between pole configurations [34]. Specific energy input for each test was calculated using the equation:

$$Specific\;energy\;(kWh/t)=\;\frac{Power\;(kW)\;\times \;Time\;(s)\;}{Mass\;(t)\;\times \;3600}$$

This formulation was used to compare energy consumption across VSI, LS-HPGR, and HS-HPGR comminution strategies.
